# Novel GIRlncRNA Signature for Predicting the Clinical Outcome and Therapeutic Response in NSCLC

**DOI:** 10.3389/fphar.2022.937531

**Published:** 2022-08-03

**Authors:** Qiangzhe Zhang, Xicheng Liu, Zhinan Chen, Sihe Zhang

**Affiliations:** ^1^ State Key Laboratory of Medicinal Chemical Biology and College of Pharmacy, Tianjin Key Laboratory of Molecular Drug Research, Nankai University, Tianjin, China; ^2^ Department of Physiology and Pathophysiology, School of Basic Medical Sciences, Capital Medical University, Beijing, China; ^3^ National Translational Science Center for Molecular Medicine, Department of Cell Biology, State Key Laboratory of Cancer Biology, Fourth Military Medical University, Xi’an, China; ^4^ Department of Cell Biology, School of Medicine, Nankai University, Tianjin, China

**Keywords:** long non-coding RNA, somatic mutation, genomic instability, prognostic signature, non–small cell lung cancer

## Abstract

**Background:** Non–small cell lung cancer (NSCLC) is highly malignant with driver somatic mutations and genomic instability. Long non-coding RNAs (lncRNAs) play a vital role in regulating these two aspects. However, the identification of somatic mutation-derived, genomic instability-related lncRNAs (GIRlncRNAs) and their clinical significance in NSCLC remains largely unexplored.

**Methods:** Clinical information, gene mutation, and lncRNA expression data were extracted from TCGA database. GIRlncRNAs were screened by a mutator hypothesis-derived computational frame. Co-expression, GO, and KEGG enrichment analyses were performed to investigate the biological functions. Cox and LASSO regression analyses were performed to create a prognostic risk model based on the GIRlncRNA signature (GIRlncSig). The prediction efficiency of the model was evaluated by using correlation analyses with mutation, driver gene, immune microenvironment contexture, and therapeutic response. The prognostic performance of the model was evaluated by external datasets. A nomogram was established and validated in the testing set and TCGA dataset.

**Results:** A total of 1446 GIRlncRNAs were selected from the screen, and the established GIRlncSig was used to classify patients into high- and low-risk groups. Enrichment analyses showed that GIRlncRNAs were mainly associated with nucleic acid metabolism and DNA damage repair pathways. Cox analyses further identified 19 GIRlncRNAs to construct a GIRlncSig-based risk score model. According to Cox regression and stratification analyses, 14 risk lncRNAs (AC023824.3, AC013287.1, AP000829.1, LINC01611, AC097451.1, AC025419.1, AC079949.2, LINC01600, AC004862.1, AC021594.1, MYRF-AS1, LINC02434, LINC02412, and LINC00337) and five protective lncRNAs (LINC01067, AC012645.1, AL512604.3, AC008278.2, and AC089998.1) were considered powerful predictors. Analyses of the model showed that these GIRlncRNAs were correlated with somatic mutation pattern, immune microenvironment infiltration, immunotherapeutic response, drug sensitivity, and survival of NSCLC patients. The GIRlncSig risk score model demonstrated good predictive performance (AUCs of ROC for 10-year survival was 0.69) and prognostic value in different NSCLC datasets. The nomogram comprising GIRlncSig and tumor stage exhibited improved robustness and feasibility for predicting NSCLC prognosis.

**Conclusion:** The newly identified GIRlncRNAs are powerful biomarkers for clinical outcome and prognosis of NSCLC. Our study highlights that the GIRlncSig-based score model may be a useful tool for risk stratification and management of NSCLC patients, which deserves further evaluation in future prospective studies.

## Introduction

Lung cancer is the leading cause of cancer death worldwide, with an approximate 1.8 million deaths each year (Sung et al., 2021). About 85% of lung cancer patients are diagnosed with non–small cell lung cancer (NSCLC), of which lung adenocarcinoma (LUAD) and lung squamous cell carcinoma (LUSC) are the two major subtypes (Herbst et al., 2018). Although clinical approaches have achieved significant advances in NSCLC treatment, the 5-year survival rate is only 25% in 2021 (Sung et al., 2021), and there is an urgent need for the identification of novel prognostic biomarkers for improved risk stratification and enhanced therapeutic efficiency of NSCLC (Gridelli et al., 2015; Chen and Dhahbi, 2021; Peng et al., 2021).

Genomic instability and somatic mutations are two hallmarks of cancer and contribute essentially to malignant transformation (Hanahan and Weinberg, 2011). As predicted by the “Mutator Phenotype” hypothesis, mutations of DNA repair genes, oncogenes, and tumor suppressor genes (TSG) can cause increased genomic instability, which drives cancer onset and progression (Negrini et al., 2010). Additionally, defects in genes controlling chromosome cohesion, mitotic kinetochore-microtubule attachment, centrosome copy number, checkpoint function, and cell-cycle regulation can accelerate the genomic instability. Beyond that, chromosomal instability such as translocations, deletions, insertions, amplifications, and inversions of large segments as well as gains or losses of whole chromosomes can also cause genomic instability (Thompson et al., 2010; Bastians, 2015). Furthermore, epigenetic modifications, like DNA modification, histone variants and modifications, nucleosome remodeling, and non-coding RNA, together play important roles in keeping genomic stability (Reis et al., 2016; Feng and Riddle, 2020). Moreover, an undesirable tumor microenvironment can also increase genomic instability (Rummel et al., 2012), of which hypoxia is a major factor (Sonugur and Akbulut, 2019). Notably, genomic instability contributes to the acquisition of multidrug resistance in malignancy (Lee et al., 2011; Osrodek and Wozniak, 2021), which frequently gives rise to poor therapeutic response and patient outcome (Lukow et al., 2021). Therefore, novel biomarkers correlated with genomic instability are critical for cancer diagnosis, treatment, and prognosis.

Emerging evidence indicates that long noncoding RNAs (lncRNAs) play vital roles in regulating genomic stability (Nair et al., 2020). Multiple studies have revealed that lncRNAs can preserve genomic stability in the process of DNA damage response and repair. For instance, a colorectal cancer-overexpressed oncogenic lncRNA, CRNDE, could reduce DNA damage and cell apoptosis after oxaliplatin treatment (Gao et al., 2017). Furthermore, lncRNA LINP1 could serve as a scaffold linking Ku80 and DNA-PKcs and consequently coordinate non-homologous end-joining pathways to enhance the repair of DNA double-strand breaks (Zhang et al., 2016). A poorly characterized lncRNA, NORAD, maintains genomic stability by sequestering the PUMILIO protein. Once NORAD was missing, PUMILIO induce chromosomal instability by hyperactively inhibiting mitosis, DNA replication, and DNA damage repair (Lee et al., 2016). These studies strongly suggest that genomic instability-related lncRNAs (GIRlncRNAs) may provide a vital molecular signature for predicting the malignant phenotype. Recently, Bao *et al* identified a genomic instability-related lncRNA signature (GIRlncSig) for improved predication of breast cancer outcome, which combined lncRNA expression and the somatic mutation profile (Bao et al., 2020). Geng and Peng have identified two sets of GIRlncSig in LUAD and early LUAD with the favorable prognostic outcome (Geng et al., 2021; Peng et al., 2021). Although these GIRlncSigs have been associated with the prognosis of particular subtypes of lung cancer, GIRlncSig-associated tools for NSCLC prognosis have yet to be established. More importantly, the clinical significance and biological function of GIRlncSig in NSCLC remain largely unexplored.

In this study, we identified 19 somatic mutation-derived GIRlncRNAs in NSCLC by using the mutator hypothesis-derived computational frame. A GIRlncSig-based risk score model with reliable prognostic performance was constructed, which can be a powerful indicator of genomic instability, immune microenvironment infiltration, therapeutic response, drug resistance, and patient stratification, thereby improving the personalized treatment of NSCLC.

## Materials and Methods

### Data Collection and Preprocessing

The overall procedure in this study is outlined as the roadmap (Supplementary Figure S1). Transcriptomic and clinical information on NSCLC (LUAD and LUSC) were downloaded from The Cancer Genome Atlas (TCGA) database *via* the UCSC Xena Browser (https://xenabrowser.net/). Somatic mutation data were downloaded from TCGA database (https://portal.gdc.cancer.gov/). Counts data were used for the transcriptome data, and the muTect version was used for the somatic mutation data. The human gtf file containing the gene symbol was downloaded from the Ensembl database (Homo_sapiens.GRCh38.99.gtf.gz; http://www.ensembl.org). Transcriptomic, somatic mutation data, and clinical information were matched according to the sample name, and samples with missing data were excluded. Finally, 975 complete samples including gene expression, mutation, patient’s survival, and other clinical variables were obtained. The mRNAs and lncRNAs of 975 samples were annotated based on the gtf file containing the gene symbol. Then, these samples were randomly distributed into training and testing sets at a ratio of 7:3 using the “caret” package in R. The training set of 683 patients was used to identify the GIRlncSig and construct the prognostic risk model. The testing set of 292 patients was used to validate the performance of our risk model. The clinical information of NSCLC patients is summarized in Table 1.

**TABLE 1:** Clinical information of NSCLC patients.

Covariate	Type	TCGA set (n = 975)	Training set (n = 683)	Testing set (n = 292)<	*p-*value^a^
Gender	Male	584 (59.9%)	417 (61.05%)	167 (57.19%)	0.2597
	Female	391 (40.1%)	266 (38.95%)	125 (42.81%)	
Age (years)	>60	698 (71.59%)	489 (71.60%)	209 (52.74%)	0.9574
	≤60	262 (26.87%)	184 (26.93%)	78 (45.55%)	
	NA	15 (1.54%)	10 (1.46%)	5 (1.71%)	
Tumor stage	Stage I-II	772 (79.18%)	541 (79.21%)	231 (79.11%)	0.8944
	Stage III-IV	192 (19.69%)	135 (19.76%)	57 (19.52%)	
	Unknown	11 (1.13%)	7 (1.02%)	4 (1.37%)	
Pathologic M	M0	723 (74.15%)	517 (75.7%)	206 (70.55%)	0.1359
	M1	31 (3.18)	23 (3.37%)	8 (2.74%)	
	MX	221 (22.67%)	143 (20.93%)	78 (26.71%)	
Pathologic N	N0	626 (64.21%)	441 (64.57%)	185 (63.36%)	0.2510
	N1-3	333 (34.15%)	228 (33.38%)	105 (35.96%)	
	NX	16 (1.64%)	14 (2.05%)	2 (0.68%)	
Pathologic T	T1-2	821 (84.21%)	571 (83.6)	250 (85.62%)	0.4256
	T3-4	151 (15.49%)	109 (15.96%)	42 (14.38%)	
	TX	3 (0.31%)	3 (0.44%)	0 (0%)	

a Chi-squared test, *p* < 0.05 means significantly different.

### Identification of GIRlncRNAs

The lncRNA expression profile and somatic mutation pattern of 975 patients were combined to identify GIRlncRNAs by a mutator hypothesis-derived computational frame (Bao et al., 2020) (Supplementary Figure S2). Briefly, NSCLC patients were ranked in increasing order according to the cumulative number of somatic mutations. The top 25% of patients with low mutation frequency were designated as the genomic stable (GS) group, and the last 25% of patients with high mutation frequency were designated as genomic unstable (GU) group. LncRNA expression profiles between the two groups were compared, and a volcano plot was made using the “edgeR” package in R. Differentially expressed (DE) lncRNAs (|Fold Change| > 1.0 and adjusted *p* < 0.05) were defined as GIRlncRNAs, and their expression levels were normalized to all patients.

### Co-Expression Network, GO and KEGG Enrichments, and Alternative Splicing Analysis

The mRNA-interacting GIRlncRNAs were extracted from the RNAInter database (http://www.rna-society.org/rnainter/). Then, the co-expression network of mRNA-interacting GIRlncRNAs was visualized by Cytoscape (V3.7.2) (Chin et al., 2014). To explore the biological function of GIRlncRNAs, GO functional enrichment and KEGG pathway analyses were performed using the “clusterProfiler,” “org.Hs.eg.db,” “enrichplot,” and “ggplot2” packages in R. Statistical significance was considered with adjusted *p*-value < 0.05. To determine the AS events associated with GIRlncRNAs, AS data of NSCLC were downloaded from LncAS2Cancer database (https://lncrna2as.cd120.com/), and integrated with corresponding GIRlncRNAs. Statistical histogram and Upset plot were drawn using “ggplot2” and “UpSetR” packages in R, respectively.

### Hierarchical Cluster Analysis

The normalized DE-lncRNAs from 975 samples were collected for hierarchical cluster analysis using the pam method, and the spearman distances were calculated using the “ConsensusClusterPlus” package in R. All samples were divided into two clusters based on the spearman distances. One cluster with low mutation counts was assigned as GS-like subtype. The other with high mutation counts were defined as GU-like subtype (Mann–Whitney *U* test, *p* < 0.05). Finally, Kaplan–Meier survival curves and the expression heatmap of DE-lncRNAs in the two subtypes were plotted using “survival,” “survminer,” and “ComplexHeatmap” packages in R.

### GIRlncRNA-Clustered Molecular Subtype and Characteristic Analyses

To estimate the differential mutation frequency between GS and GU-like subtypes of NSCLC patients, driver genes from Cancer Gene Census (CGC) catalog in the COSMIC database (https://cancer.sanger.ac.uk/census) were downloaded. Fifty-four driver genes with corresponding MAF files were extracted and determined their mutation frequency. The landscape of mutation frequency was drawn using the “maftools” package in R. Furthermore, the differential expression of driver genes from two NSCLC subtypes was analyzed using “edgeR” package in R. The cutoff criteria were |log2 FC|>1 and adjusted *p* < 0.05. The Wilcoxon test was used to analyze the differentially expressed GIRlncRNAs. Moreover, the immune, stromal, and ESTIMATE scores for the two NSCLC subtypes were determined by the “estimate” package in R. In addition, tumor mutation burden (TMB) data were downloaded from TCGA database (https://gdc.cancer.gov/) and TMB scoring for the two NSCLC subtypes was performed. Finally, the scores for the mRNA expression-based stemness index (mRNAsi) were calculated, and NSCLC patients were stratified into different molecular subtypes by referring to the matrix in Malta et al. (2018). Boxplots for GS- and GU-like groups were drawn using the “ggpubr” package in R (Student t-test, adjusted *p* <0.05).

### Identification of GIRlncSig and Performance Evaluation

To estimate the correlation of GIRlncRNA expression with overall survival (OS) of NSCLC patients, a univariate Cox proportional hazards regression analysis was performed in the training set by using “survival” and “survminer” packages in R. The candidate GIRlncRNAs were screened with *p* < 0.01. Then, the least absolute shrinkage and selection operation (LASSO) Cox regression analysis was carried out to identify the GIRlncRNAs with the most robust prognostic values. Finally, the resulting GIRlncRNAs were collected to construct a GIRlncSig based on the weighted expression level and coefficient (coef) from LASSO regression analysis. The risk score formula for GIRlncRNAs was calculated as follows:

$${\text{GIRlncSig Risk Score}}_{i}=\;\overset{n}{\underset{j=1}{\sum }}\exp_{ji}\times {\text{β}}_{j}.$$

The “*exp*” and “*β*” represent the expression level and coefficient of each prognostic lncRNA, respectively. GIRlncSig risk score represents the sum of expression level of each prognostic lncRNA multiplied by the coef of corresponding GIRlncSig. *i* represents the sample, and *j* represents the prognostic lncRNA. Based on the risk score for all samples, high and low-risk patients were then recognized by using the median value as the cut-off point. Evaluation analyses, including the Kaplan–Meier survival curve, receiver operating characteristic curve (ROC), risk distribution, survival status of all patients, and heatmap of selected GIRlncSig expression profile were applied to test the predication performance of our risk model in training, testing, and TCGA sets.

### Independent Prognostic and Clinical Stratification Analysis

To test whether the GIRlncSig risk score could be potentiated as a prognostic factor independently from other clinical variables (age, gender, and tumor stage), univariate and multivariate Cox regression analyses (UCRA and MCRA) were performed. All variables with independent prognostic values were selected from TCGA database when their *p*-values were less than 0.05. To test the prognostic stability of GIRlncSig scoring, a clinical stratification analysis was conducted. Patients were first divided into subgroups according to clinical variables, including age (≤60 and >60), gender (female and male), pathologic T (T1-T2 and T3-T4), and tumor stage (I-II and III-IV). Second, patients in each subgroup were ranked according to their GIRlncSig risk score, and assigned to high or low-risk subgroups by referring to the median value. Finally, the survival difference between high and low-risk subgroups was calculated (log-rank test, *p* < 0.05).

### Correlation Analysis Between GIRlncSig and Drive Genes or the Microenvironment

Expression profiles of GIRlncSig and driver genes of NSCLC were extracted, and their correlation was calculated using “ggplot2” and “ggpubr” packages in R. The cutoff criteria were R > 0.3 and *p* < 0.05, respectively. Correlation between GIRlncSig score and immune, or stromal, or ESTIMATE, or mRNAsi or TMB scores were further calculated (*p* < 0.05).

### Immune Cell Infiltration, Checkpoint Inhibitor-Related Genes, and Therapeutic Response

The mRNA expression matrix of each patient was converted to 22 types of immune cell matrix with cutoff criteria of *p* < 0.05. Tumor-infiltrating immune cells include naive B cells, memory B cells, plasma cells, CD8^+^ T cells, naive CD4^+^ T cells, resting memory CD4^+^ T cells, activated memory CD4^+^ T cells, follicular helper T cells, regulatory T cells (Tregs), gamma delta T cells, resting dendritic cells, activated dendritic cells, monocytes, macrophages M0, M1, M2, resting natural killer (NK) cells, activated NK cells, resting mast cells, activated mast cells, eosinophils, and neutrophils. Based on their association with clinical outcomes, NSCLC patients were classified into high- and low-risk groups using the Cell type Identification by Estimating Relative Subsets of RNA Transcript (CIBERSORT) algorithm (Chen et al., 2018). The type and distribution of infiltrating immune cells were analyzed by “ggplot2” package in R, and presented as barplots and boxplots. Subsequently, we analyzed the expression profile of immune checkpoint inhibitor genes in high and low-risk groups. A significant difference was determined by using the Wilcoxon test with *p*-value < 0.05. Based on the functional enrichment of gene expression, SubMap module from GenePattern was used to map and merge two datasets with different traits. This module can eliminate the batch effect and predict the possible traits that are not included in the original dataset. To predict the therapeutic response by checkpoint inhibitor blocking, SubMap module was used to map the high and low-risk groups based on the therapeutic information. Prediction of the response to CTLA4 and PD1 inhibitors was particularly studied in high and low-risk NSCLC patients. The *p*-value was corrected by Bonferroni to increase the predictive sensitivity. Finally, we used R package “pRRophetic” to predict chemotherapeutic responses of each sample based on the genomics of drug sensitivity in the cancer (GDSC) database. The half-maximal inhibitory concentration (IC50) of each sample was calculated using ridge regression (*p* < 0.001). The Spearman correlation (Cor) between the RS score and IC50 to particular drugs was calculated and the significant correlations were cutoff with | Cor | > 0.1 and *p* < 0.001.

### Gene Set Variation Analysis in High and Low-Risk Groups

GSVA analysis was conducted to reveal the differential pathways between high and low-risk patients. Functional enrichment analysis was conducted based on the distinct GIRlncSig patterns extracted from KEGG and MSigDB database using “GSVA” package in R. Significant differential signal pathways between high and low-risk groups were extracted by “limma” package with the threshold for adjusted *p* < 0.05 and |logFC|>0.1. Visualized heatmap of KEGG pathway was plotted using “ComplexHeatmap” package in R.

### Construction and Validation of the Nomogram Score System

To develop a nomogram score system for NSCLC patients, MCRA was performed to extract the powerful OS predictors. To visualize the results of Cox regression and predict the survival of NSCLC patients, the prognostic nomogram was plotted by using “rms” and “survival” packages in R. First, the Cox proportional hazards regression model was constructed by using the cph () function, and the survival () function was used to calculate the survival probability. Finally, the nomogram () function was used to create the nomogram, time-dependent ROC, and calibration curves of OS. The predictive performance of the nomogram was validated by calibration curves, ROC, and decision curve analysis (DCA). The area under curves (AUCs) of the nomogram for predicting 1-, 3-, 5-, and 10-year OS of NSCLC patients were plotted.

### Statistical Analyses

The chi-squared test, Student’s t-test, Wilcoxon test, and Mann–Whitney *U*-test were employed to examine the differential variables from datasets or groups. Statistical significance was considered as *p* < 0.05. R (version 3.6.3) was used to perform all statistical analyses, and the results were visualized by corresponding functional packages.

## Results

### Identification of GIRlncRNAs for NSCLC Patients

**FIGURE 1:**
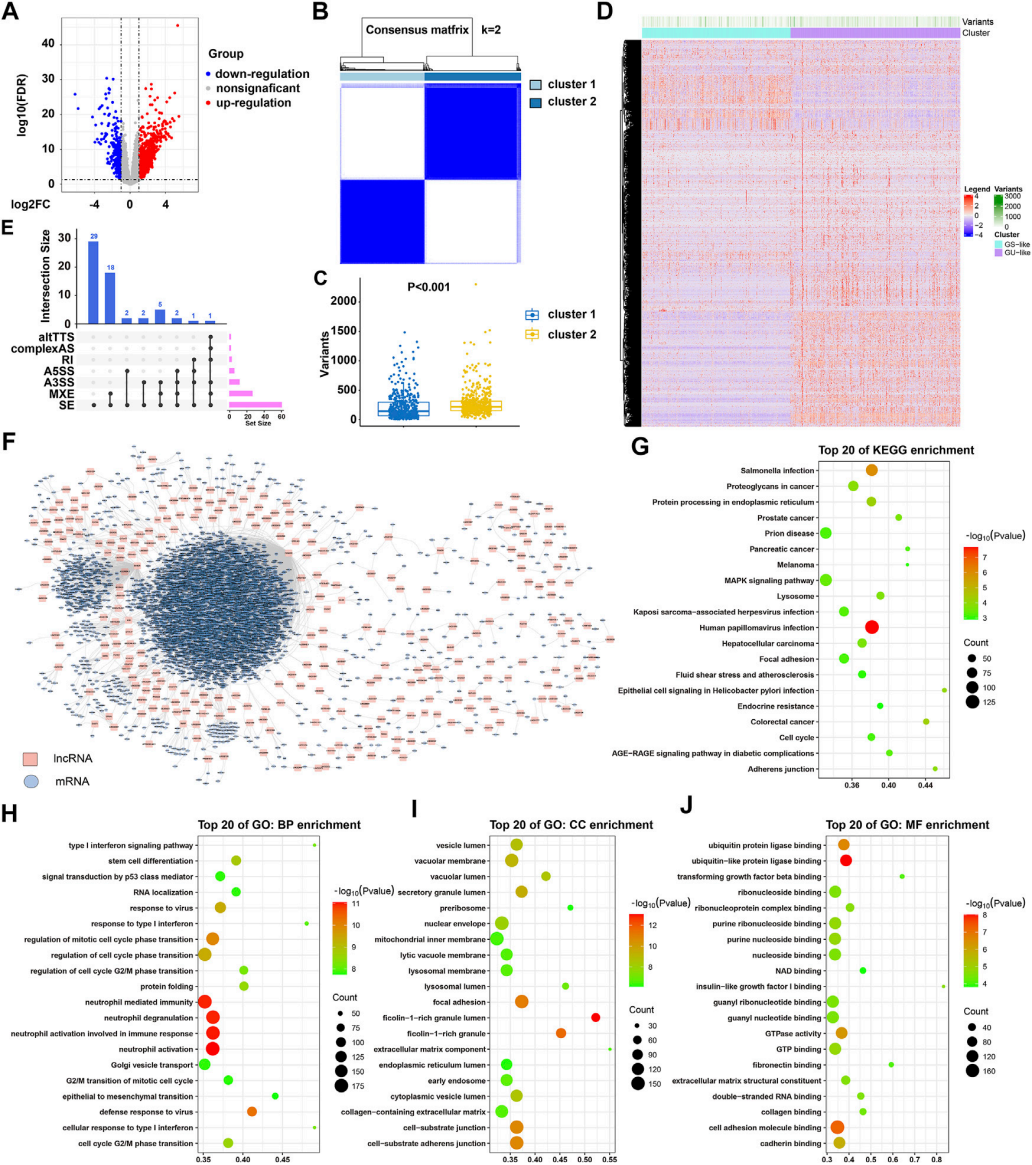
Identification and functional analysis of GIRlncRNAs for NSCLC patients. **(A)** Volcano plot of the GIRlncRNA distribution. 1,446 DE-lncRNAs between the GU group (*n* = 244) and GS group (*n* = 244) are shown. **(B)** Unsupervised clustering of 975 NSCLC patients according to the expression pattern of identified GIRlncRNAs. Cluster 1, light blue box. Cluster 2, dark blue box. **(C)** Mutation frequency analysis of DE-lncRNAs in two clusters derived from unsupervised clustering analyses. Cluster 1, blue scatterplot. Cluster 2, orange scatterplot. **(D)** Expression heatmap of DE-lncRNAs from 975 NSCLC patients. Subtype classification was performed by unsupervised hierarchical clustering analysis. Cluster 1 (cyan box) is designated as the GS-like group, and cluster 2 (orchid box) is designated as the GU-like group. **(E)** Upset plotting the statistical AS events of 60 DE-lncRNAs. **(F)** Co-expression network of mRNA-interacting GIRlncRNAs based on Pearson correlation coefficient. **(G)** Enriched KEGG pathways for the mRNA-interacting GIRlncRNAs. **(H–J)** Enriched GO pathways for the mRNA-interacting GIRlncRNAs in three parts: BP **(H)**, CC **(I)**, and MF **(J)**.

Following TCGA data collection, annotation, and preprocessing, 975 samples were obtained. These samples were subsequently ranked by their gene mutation counts. The top 25% samples (*n* = 244) with a low mutation frequency were designated as the GS group, and the last 25% samples (*n* = 244) with a high mutation frequency were designated as the GU group (Supplementary Figure S2; Supplementary Table S1). A total of 1,446 DE-lncRNAs between these two groups were identified from an expression matrix containing 10,480 lncRNAs (|log2 FC|>1, FDR adjusted *p* < 0.05), among which 1138 lncRNAs were upregulated and 308 lncRNAs were downregulated (Figure 1A; Supplementary Table S2). Based on the expression profile of these DE-lncRNAs, all samples were clustered under an unsupervised hierarchical clustering analysis by “ConsumusClusterPlus” package. Two clusters were obtained as shown in the heatmap (Figure 1B; Supplementary Table S3). The resulting subtypes were positively correlated with the mutation frequency (R = 1.22; *p* = 0.0001) and the mutation frequency in cluster 2 was significantly higher than that in cluster 1 (Figure 1C). Therefore, cluster 1 and cluster 2 were named as GS and GU-like subtypes, respectively. As shown by the heatmap of DE-lncRNAs, a significant expression difference was observed between these two subtypes (Figure 1D). These results identified 1,446 lncRNAs as candidates for GIRlncRNAs of NSCLC.

Further analyses found that 60 candidate GIRlncRNAs can undergo AS events, of which all DE-lncRNAs contained skipped exon (SE), and 26 DE-lncRNAs contained mutually exclusive exons (MXE) (Figure 1E; Supplementary Figure S3; Supplementary Table S4). Co-expression network analyzing the interaction of DE-lncRNA with mRNA resulted in 4912 interaction pairs (Figure 1F; Supplementary Table S5), among which 3,802 (77.4%) mRNAs and 334 (6.8%) mRNAs were respectively interacting with lncRNA FENDRR and BANCR. It was also found that 38 and 5 lncRNAs were, respectively, interacting with mRNA AR and TNPO2 (Supplementary Table S6). GO enrichment and KEGG pathway analyses showed that these mRNA-interacting GIRlncRNAs were significantly enriched in a nucleoside or ribonucleoside binding, nuclear membranes enveloping, cell-cycle checkpoint, tumorigenesis, and virus infection signaling pathways (Figures 1G–J; Supplementary Tables S7, S8 **)**. These results suggested that the identified mRNA-interacting GIRlncRNAs may be essential for regulating genomic stability.

### Characteristics of GIRlncRNA-Clustered Molecular Subtypes in NSCLC Patients

**FIGURE 2:**
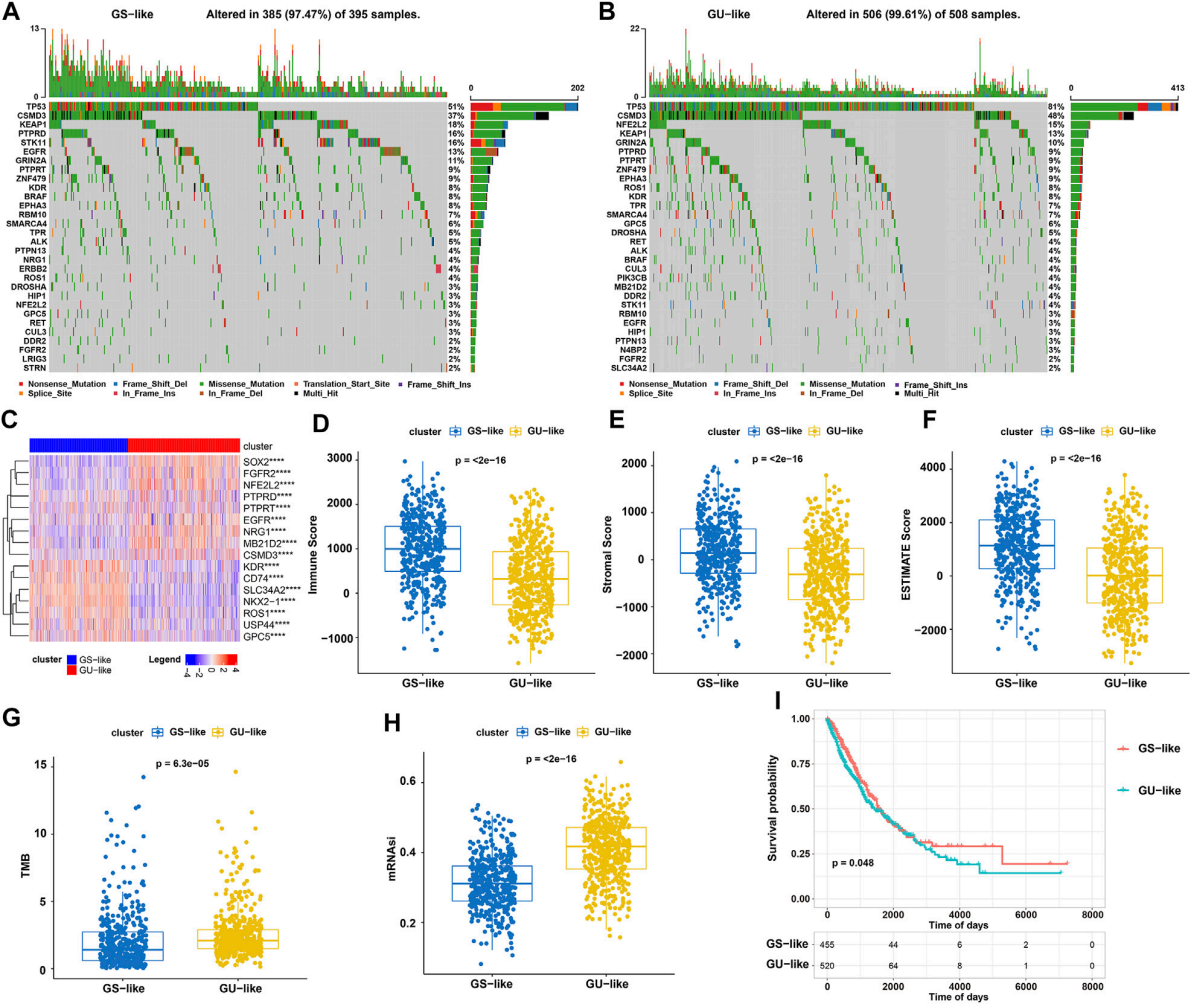
Mutation and expression landscapes of driver genes, tumor microenvironment assessment, and Kaplan–Meier survival analyses. **(A,B)** Mutation frequency of 54 driver genes (top 30) in 395 GS-like **(A)** and 508 GU-like **(B)** NSCLC patients. Each column represents an individual patient. The upper barplot indicates TMB, and the number on the right indicates the mutation frequency for each regulator. The right barplot shows the proportion of each variant type. **(C)** Expression landscape of 17 driver genes differently expressed between GS and GU-like patients. **(D–H)** Immune **(D)**, stromal **(E)**, and ESTIMATE **(F)** score and TMB **(G)** and mRNAsi **(H)** analyses in GS and GU-like groups. **(I)** Survival analysis of GS and GU-like groups of NSCLC patients.

Investigating the mutation profile of driver genes showed that GU-like subtype patients exhibited higher mutation frequency than that in GS-like subtype patients. Being the most frequent mutation genes in NSCLC, TP53 and CSMD3 were mutated in 81% and 48% of GU-like subtype patients, respectively. However, these two gene mutations only occurred respectively in 51% and 37% of GS-like subtype patients (Figures 2A,B). Further expression analysis of 54 driver genes showed that 16 genes were differentially expressed between GS and GU-like subtypes, among which nine genes were upregulated and seven genes were downregulated in GU-like patients (Figure 2C). Tumor microenvironment scoring results showed that the immune, stromal and ESTIMATE scores in GU-like subtypes were significantly lower than those in GS-like subtypes (Figures 2D–F). Further analysis of NSCLC tumor characteristics showed that TMB and mRNAsi scores in GU-like subtypes were significantly higher than those in GS-like subtypes (Figures 2G,H). Moreover, Kaplan–Meier survival analysis showed that the survival of GS-like patients was significantly better than that of GU-like patients (Figure 2I). These data indicated that NSCLC patients with the GU-like molecular subtype have more aggressive tumors than those with GS-like molecular subtypes.

### Identification and Evaluation of GIRlncSig for NSCLC Patients

**FIGURE 3:**
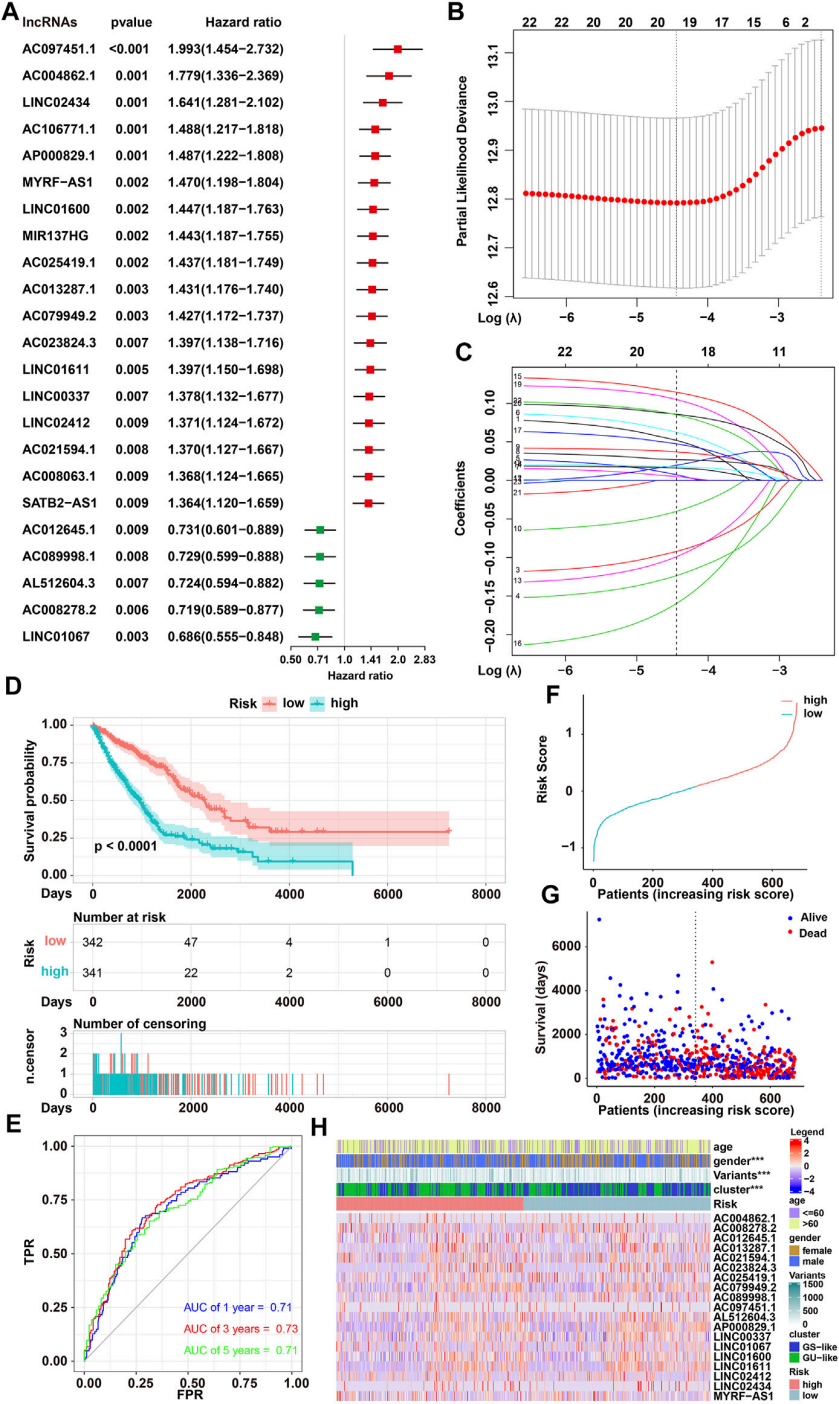
Identification of the GIRlncSig and its predictive performance in a training set. **(A)** Forest plot of OS-associated GIRlncRNAs based on the univariate Cox proportional hazards regression analysis. Five GIRlncRNAs acted as protective risk factors for patients’ survival (green), while 18 GIRlncRNAs acted as prognostic risk factors (red). **(B)** Distribution plot of partial likelihood deviation based on LASSO regression analysis. Nineteen GIRlncRNAs were selected when log lambda was equal to −4.4 (the minimum). **(C)** Distribution plot of LASSO coefficient (log lambda = −4.4). **(D)** Kaplan–Meier survival curves of low and high-risk NSCLC patients predicted by the GIRlncSig (log-rank test, *p* < 0.0001). **(E)** ROC of NSCLC patients at 1, 3, or 5 years predicted by GIRlncSig. **(F,G)** Risk distribution **(F)** and survival status **(G)** of NSCLC patients. **(H)** Expression heatmap of selected GIRlncRNAs.

Univariate Cox proportional hazards regression analysis showed that the expressions of 23 GIRlncRNAs were significantly associated with OS of NSCLC patients in our training set (*p* < 0.01 Figure 3A; Supplementary Table S9). LASSO regression analysis was performed to identify the GIRlncRNAs with more significant associations (*p* < 0.05). When the partial likelihood deviation reached to the minimum (Log Lambda = −4.4), 19 GIRlncRNAs were screened out and used to construct a risk model for survival prediction (Figures 3B,C; Supplementary Table S10). To assess the prognostic risk of NSCLC patients, a GIRlncSig was created based on the expression level of 19 GIRlncRNAs and the coefficients from LASSO analysis. GIRlncSig score = LINC01067* (−0.1595) + AC012645.1 * (−0.1235) + AL512604.3 * (−0.0989) + AC008278.2 * (−0.0921) + AC089998.1 * (−0.0401) + AC023824.3 * 0.0050 + AC013287.1 * 0.0073 + AP000829.1 * 0.0159 + LINC01611 * 0.0177 + AC097451.1 * 0.0197 + AC025419.1 * 0.0270 + AC079949.2 * 0.0369 + LINC01600 + 0.0474 + AC004862.1 * 0.0518 + AC021594.1 * 0.0623 + (MYRF–AS1) * 0.0851 + LINC02434 * 0.0858 + LINC02412 * 0.1049 + LINC00337 * 0.1143. In the formula of GIRlncSig, 14 GIRlncRNAs (AC023824.3, AC013287.1, AP000829.1, LINC01611, AC097451.1, AC025419.1, AC079949.2, LINC01600, AC004862.1, AC021594.1, MYRF–AS1, LINC02434, LINC02412, and LINC00337) with positive coefficients were designated as risk factors. In contrast, five lncRNAs (LINC01067, AC012645.1, AL512604.3, AC008278.2, and AC089998.1) with negative coefficients were designated as protective factors. Either upregulated expression of prognostic GIRlncRNAs or downregulated expression of protective GIRlncRNAs was significantly related to decreased OS of NSCLC patients. Based on the GIRlncSig score (cutoff = 0.075), 341 patients with a high score were classified into a high-risk group, and 342 patients with a low score were classified into a low-risk group (Supplementary Table S11). Kaplan–Meier survival analysis showed that the survival of low-risk NSCLC patients was significantly better than that in high-risk patients (*p* < 0.0001, log-rank test; Figure 3D). The AUCs of time-dependent ROC in the training set were 0.71, 0.73, 0.71 for 1-, 3-, 5-year survival, respectively, as predicted by GIRlncSig (Figure 3E). Finally, the risk distribution and survival status of NSCLC patients together with the expression heatmap of 19 GIRlncRNAs in the training set were plotted (Figures 3F–H). The results showed that high-risk-scored patients with such a GIRlncSig were mainly derived from the GU-like group and exhibited shorter survival. All these results strongly supported the utility and effectiveness of our GIRlncSig in predicting NSCLC prognosis.

**FIGURE 4:**
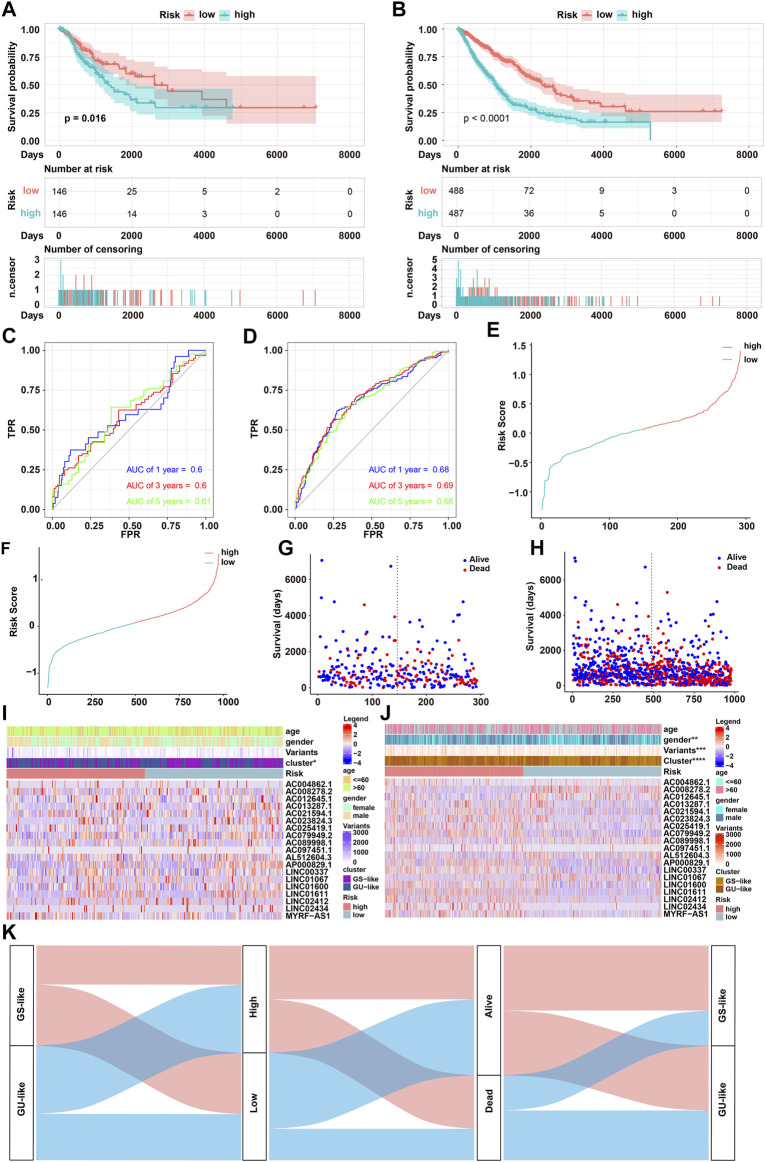
Performance evaluation of GIRlncSig in testing and TCGA sets. **(A,B)** Kaplan–Meier survival curves of GIRlncSig-predicted low- or high-risk NSCLC patients in testing [**(A)**, log-rank test; *p* < 0.05] and TCGA [**(B)**, log-rank test, *p* < 0.0001] sets. **(C,D)** ROC of 1-, 3-, or 5- year survival predicted by GIRlncSig in testing **(C)** and TCGA **(D)** sets. **(E–J)** Risk distribution, survival status of patients, and the expression profile of GIRlncSig in testing **(E**,**G**,**I)** and TCGA **(F,H,J)** sets. **(K)** Sankey diagram for the distribution of NSCLC patients in GS or GU-like groups, low or high-risk groups, and dead or alive groups.

To evaluate the robustness of our GIRlncSig, its prognostic performance in two independent data sets was further tested. A total of 392 NSCLC patients from the testing set and 975 NSCLC patients from the TCGA set were classified into high- and low-risk groups based on the GIRlncRNA risk score (Supplementary Table S11). Kaplan–Meier survival curves in the testing set (*p* < 0.016, Figure 4A) and entire TCGA set (*p* < 0.0001, Figure 4B) showed that patients from the low-risk group had better survival outcomes than patients from the high-risk group. The AUCs of time-dependent ROC in the testing set were 0.6, 0.6, and 0.61 for the 1-, 3-, and 5-year survival predicted by the GIRlncSig, respectively (Figure 4C). Similar results were obtained in TCGA set, where the AUCs of ROC were overall approximately 0.7 (Figure 4D). Furthermore, risk distribution, and survival of patients together with GIRlncSig expression heatmap in both sets showed that high-risk scored patients were mainly derived from the GU-like group and exhibited shorter survival (Figures 4E–J). Additionally, the Sankey diagram also showed that high-risk patients accounted for a higher proportion of mortality in the GU-like group, while these patients accounted for a less proportion of mortality in the GS-like group (Figure 4K). Together, these results further support that our GIRlncSig can predict the prognosis of NSCLC patients.

### Correlation of GIRlncSig With the Aggressiveness of NSCLC

**FIGURE 5:**
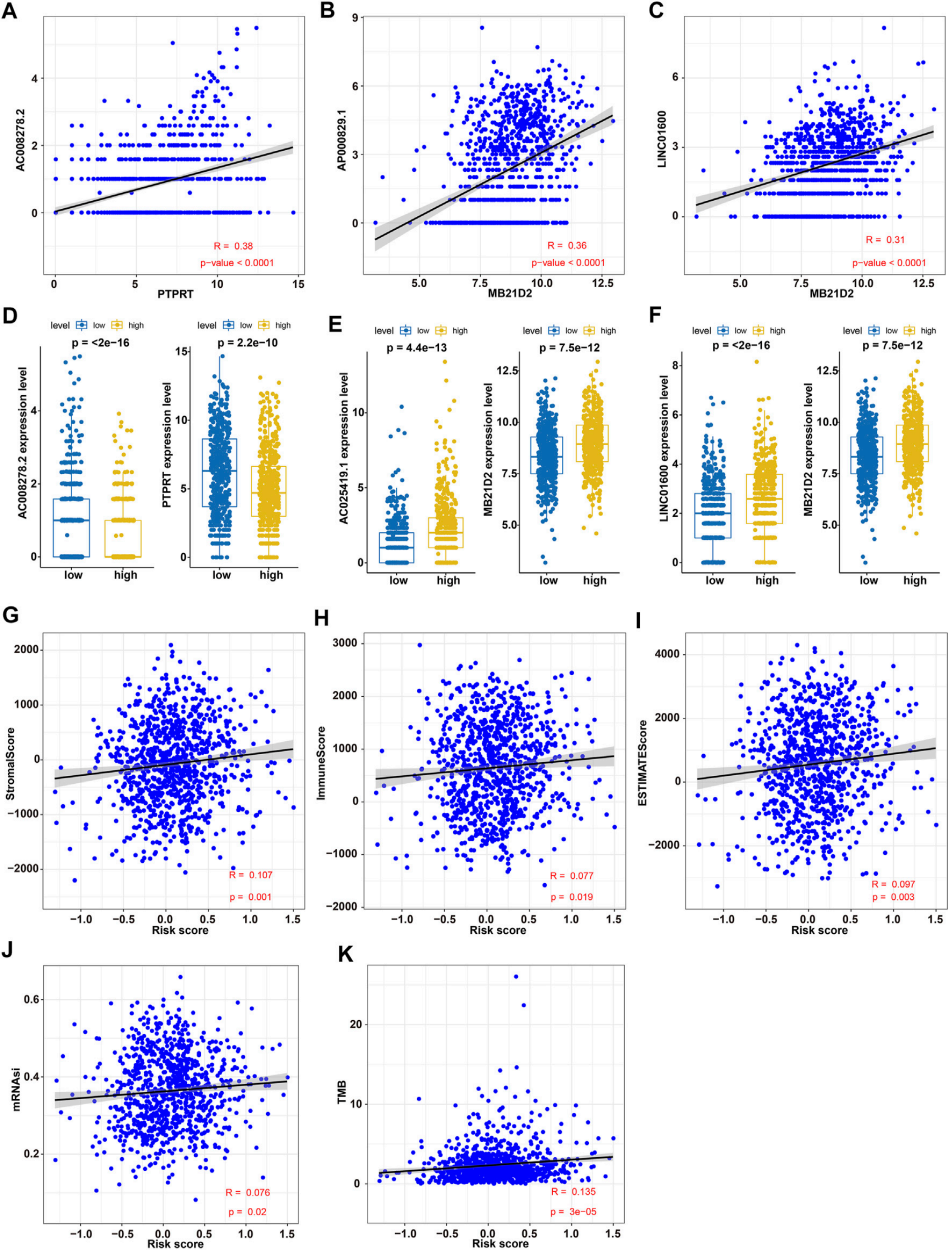
Correlation analyses between GIRlncSig and malignant characteristics of NSCLC. **(A–C)** Pearson correlation analysis of GIRlncRNA-driver gene regulatory pairs: AC008278.2 and PTPRT **(A)**, AP000829.1 and MB21D2 **(B)**, LINC01600 and MB21D2 **(C)**; **(D–F)** Differential expression analysis of correlated regulatory pairs in different risk groups: AC008278.2 and PTPRT **(D)**, AP000829.1 and MB21D2 **(E)**, and LINC01600 and MB21D2 **(F)**. **(G–K)** Pearson correlation analysis between the risk score and immune score **(G)**, stromal score **(H)**, ESTIMATE score **(I)**, mRNAsi score **(J)**, or TMB score **(K)** in NSCLC patients.

Since GIRlncSig possessed a robust prognostic performance, the correlation analysis between identified GIRlncRNAs and differentially expressed driver genes was further performed. Three positively correlated regulatory pairs (AC008278.2 and PTPRT, AP000829.1 and MB21D2, LINC01600 and MB21D2) were screened out by Pearson correlation analysis (R > 0.3 and *p* < 0.05) (Figures 5A–C; Supplementary Table S12). The expression patterns of these correlated pairs were consistent in high- and low-risk groups (Figures 5D–F). To determine the relationship of GIRlncSig with tumor microenvironment characteristics, Pearson correlation coefficients between GIRlncSig risk score and immune, or stromal, or ESTIMATES, or mRNAsi, or TMB scores were calculated separately. The results of correlation analysis showed that GIRlncSig-based risk scores were positively correlated with all characteristics of the tumor microenvironment (Figures 5G–K). Notably, the strongest correlation of the risk score with TMB was observed (R = 0.135; Figure 5K). Further correlation analyses between the GIRlncSig and infiltrating immune cells were performed. Although the resulted NSCLC-infiltrated immune cell types were different depending on algorithms, similar trends of cell distribution were as follows: high infiltrating level of B cell (especially memory B cell) and T cell (especially CD4^+^ and CD8^+^ memory T cells) populations were positively associated with the expression of five protective lncRNAs. By contrast, infiltrating B and T cells presented an opposite association trend for fourteen risk lncRNAs (Supplementary Figures S4–S10). High infiltrating levels of macrophages (especially M1 and M2), neutrophils, monocytes, and myeloid dendritic cells were positively associated with the expression of protective lncRNAs but negatively associated with the expression of risk lncRNAs (Supplementary Figures S4–S10). These results suggested that GIRlncSig was significantly correlated with the malignancy of NSCLC.

### Risk Stratification of NSCLC Patients With GIRlncSig Score and Clinical Variables

**FIGURE 6:**
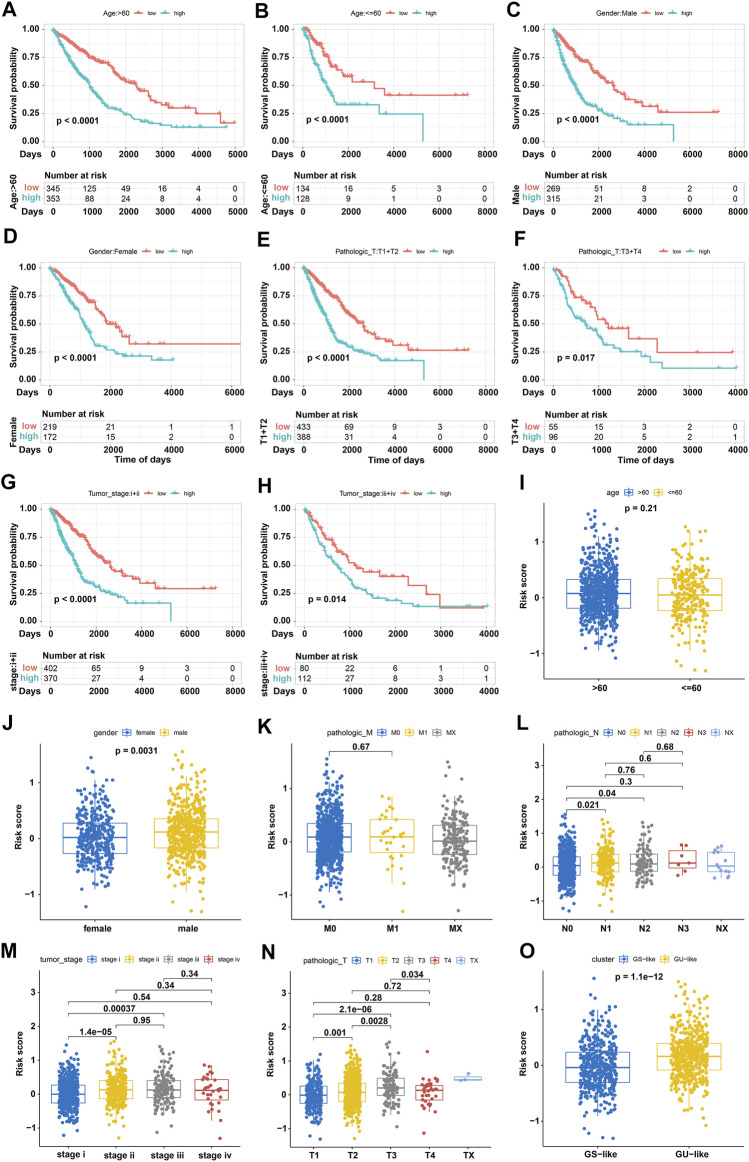
Risk stratification analyses based on GIRlncSig score and clinical variables. **(A–H)** Kaplan–Meier survival curves of high and low-risk subgroups for old **(A)**, young **(B)**, male **(C)**, female **(D)**, T1-2 stage **(E)**, T3-4 stage **(F)**, stage I-II **(G)**, and stage III-IV **(H)** patients. **(I**–**N)** Boxplots showing GIRlncSig-derived risk scores stratified by age **(I)**, gender **(J)**, and pathologic M **(K)**, N **(L)**, T **(M)**, and tumor stage **(N)** in NSCLC patients. **(O)** Boxplot of GIRlncSig-derived risk scores in GS- and GU-like NSCLC groups.

To validate the stability of our score model, a risk stratification analysis was conducted to determine the prognostic performance of GIRlncSig. NSCLC patients were first grouped based on their clinical parameters, then stratified into subgroups by GIRlncSig-derived risk score. Kaplan–Meier survival analyses showed that patients with low-risk scores had better survival outcomes than those with high-risk scores in all stratified subgroups (*p* < 0.05; Figures 6A–H). The distribution of risk scores for all stratified subgroups, including age, gender, pathologic M, pathologic N, pathologic T, tumor stage, and GS/GU-like groups, was further determined. No difference in the distribution of risk score between young and old NSCLC patients was observed (*p* < 0.05; Figure 6I). However, the risk score distribution of male patients was significantly higher than that of female patients (*p* < 0.05; Figure 6J), and the risk score distribution of the GU-like group was significantly higher than that in the GS-like group (*p* < 0.05; Figure 6O). For the groups of pathologic N, T, and tumor stage but not pathologic M, the risk score for the primary stage was always low. Interestingly, higher risk scores were frequently observed in patients with advanced stage (*p* < 0.05; Figures 6K–N). These results highlight the stability of our GIRlncSig-based risk score model.

### Therapeutic Evaluation of NSCLC Patients by the GIRlncSig Score

**FIGURE 7:**
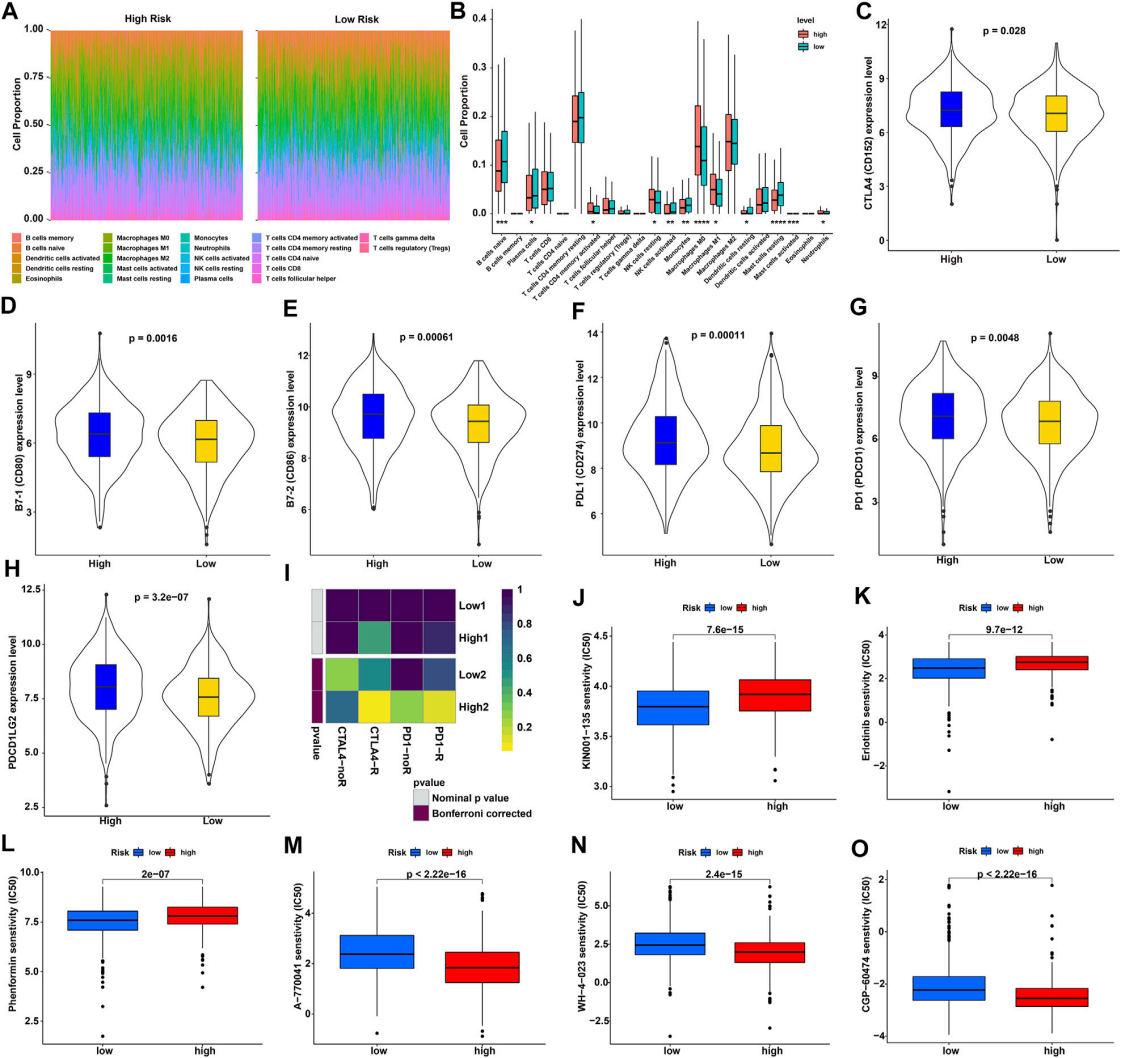
Immune evaluation and drug resistance analyses in high- and low-risk groups of NSCLC patients. **(A)** Distribution of 22 types of immune cells in high- and low-risk groups of NSCLC patients. **(B)** Boxplot of differentially infiltrated immune cells in high- and low-risk groups of NSCLC patients (*p* < 0.05). **(C–H)** Violin plot of the differentially expressed checkpoint genes in high- and low-risk NSCLC patients. **(I)** Immunotherapy response predicted for high- and low-risk NSCLC patients by SubMap module. **(J-O)** Chemotherapeutic sensitivity predicted for high- and low-risk NSCLC patients by the pRRophetic package.

The abundance of tumor-infiltrating immune cells and the expression profile of immune checkpoint genes have a strong impact on tumor treatment, so we carried out immune cell infiltration analysis with the CIBERSORT algorithm to evaluate our GIRlncSig. Abundance ratios and differential boxplots of 22 types of immune cells in high and low-risk NSCLC patients were plotted (Figure 7A; Supplementary Table S13). The abundance ratios of infiltrated M0, M1 macrophages, and resting NK cells in the high-risk score group were significantly higher than those in low-risk score group. However, naive B cells, plasma cells, monocytes, and resting mast cells in the high-risk score group were remarkably lower than those in the low-risk score group (Figure 7B). Subsequently, the expression profile of immune checkpoint genes, CTLA4 (CD152), B7-1 (CD80), B7-2 (CD86), PDL1 (CD274), PD1 (PDCD1) and PDL2 (PDCD1LG2), in high and low-risk groups was further analyzed (Figures 7C–H). The violin plots showed a significant difference in the expression levels of CTLA4, CD80, CD86, CD274, PDCD1, and PDCD1LG2 between low and high-risk groups (Figures 7C–H). Therefore, the SubMap module in GenePattern database was further employed to predict the risk score effect on immunotherapy of NSCLC patients. Results of the corrected Bonferroni analysis suggested that the patients in the high-risk group were slightly more sensitive to CTLA4 and PD1 inhibitors than those in the low-risk group (*p* = 0.0619 and 0.0739; Figure 7I). We also evaluated the chemotherapeutics sensitivity in different risk groups predicted by pRRophetic package. It was found that a total of 76 drugs, including four resistant drugs and 72 sensitive drugs, were correlated with the RS scores (Supplementary Figure S11). The predicted results showed that NSCLC patients in the high-risk group were more resistant to KIN001-135, erlotinib and phenformin than those in the low-risk group. However, these high-risk NSCLC patients were more sensitive to A-770041, WH-4-023, and CGP-60474 (Figures 7J–O). Overall, these results suggested that GIRlncSig could be used for the evaluation of immune cell distribution and immunotherapy response in NSCLC patients.

### Genomic Instability-Related Signal Pathways Were Enriched in High-Risk Patients

**FIGURE 8:**
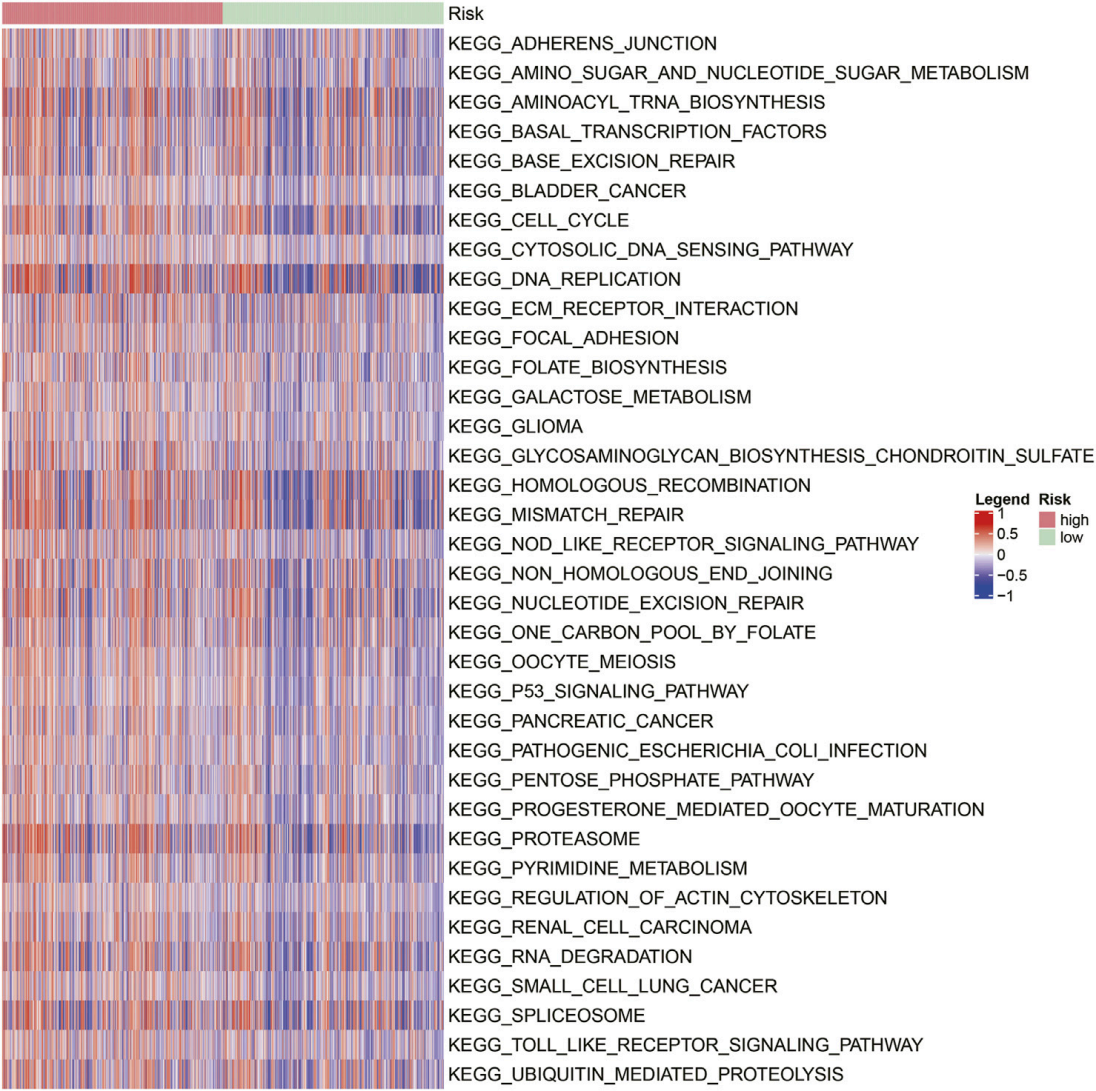
Heatmap of KEGG pathway enrichment in high- and low-risk groups of NSCLC patients.

To explore the biological function associated with the GIRlncSig, functional enrichment analysis was conducted for high and low-risk groups of NSCLC patients using “GSVA” package. MSigDB database-based KEGG analysis revealed that 32 differentially enriched items were significantly enriched in the high-risk scored group (Figure 8). Notably, two types of signaling pathways were markedly enriched in the high-risk scored group. One was the nucleic acid metabolic pathway including pyrimidine metabolism, folate biosynthesis, DNA replication, and RNA degradation. The other was the DNA damage repair pathway including mismatch repair, base excision repair, nucleotide excision repair, non-homologous end joining, and homologous recombination. Notably, these two types of pathways were strongly associated with genomic stability.

### Independent Prognostic Evaluation and Nomogram Construction Based on the Risk Score and Clinical Variables

**FIGURE 9:**
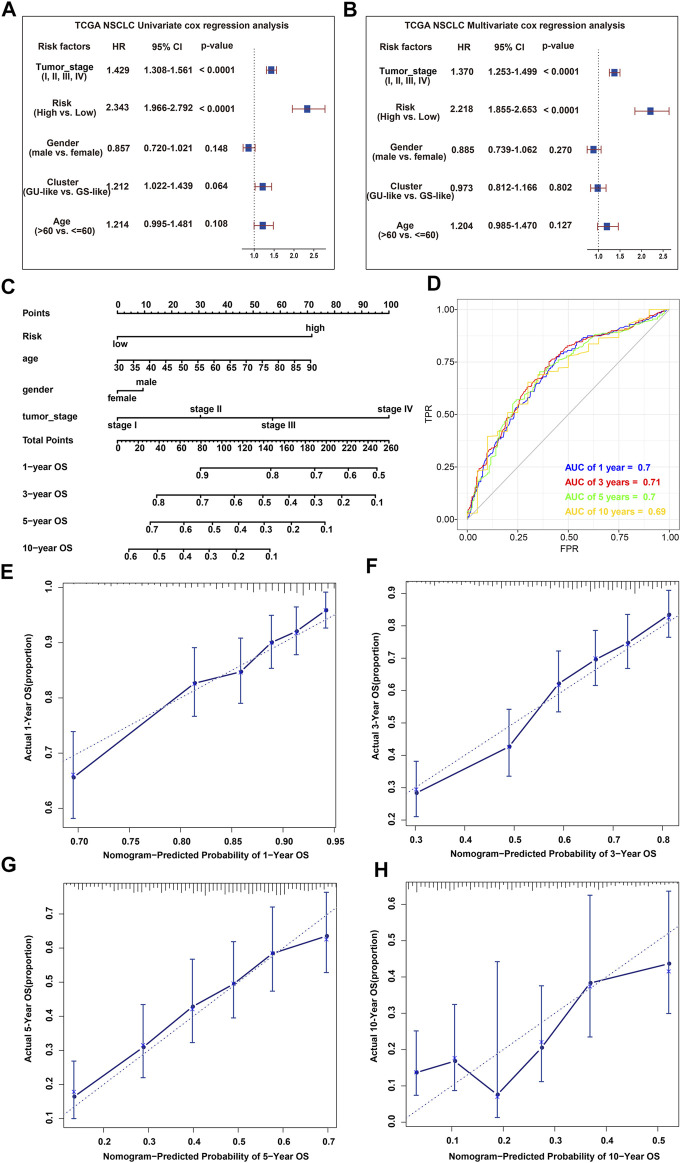
Independent prognostic evaluation and nomogram construction. **(A,B)** UCRA **(A)** and MCRA **(B)** based on GIRlncSig-based risk scores together with clinical variables; **(C)** MCRA-developed nomogram for predicting 1-, 3-, 5-, and 10-year survival of NSCLC patients; **(D)** MCRA-developed ROC for predicting 1-, 3-, 5-, and 10-year survival of NSCLC patients; **(E–H)** Calibration curves for predicting 1- **(E)**, 3- **(F)**, 5- **(G)**, and 10- **(H)** year survival of NSCLC patients.

To verify whether GIRlncSig was an independent prognostic factor, UCRA was conducted on variables including clinical variables and GIRlncSig-based risk score. Then, MCRA was used to evaluate the prognosis of all included variables. The UCRA and MCRA results showed that the GIRlncSig-based risk score and tumor stage exhibited good prognostic performance (*p* < 0.0001; Figures 9A,B; Supplementary Table S14). Other variables, such as cluster, age, and gender, had no significant correlation with the OS of NSCLC patients (*p* > 0.05). These results suggested that the prognostic value of GIRlncSig was independent of other clinical variables. To test its clinical utility, a statistical nomogram was created by integrating GIRlncSig-based risk score with TCGA clinical information (age, gender, and tumor stage) (Figure 9C). The C-index of nomogram was 0.67, and the AUCs of ROC for 1-, 3-, 5-, and 10-year survival predictions were 0.69, 0.71, 0.70, and 0.69, respectively (Figure 9D). The calibration plots of 1-, 3-, 5-, and 10-year OS showed good agreements between the actual survival rate and the nomogram-predicted survival rate (Figures 9E–H). Therefore, these data suggested that the nomogram has a good prediction performance and could provide clues for clinical diagnosis of NSCLC.

## Discussion

Genomic instability is an evolving hallmark of most cancers (Hanahan, 2022). It is also a major driver of carcinogenesis, drug sensitivity, tumor-microenvironment-shaping, and immune contexture in NSCLC (Raynaud et al., 2018; Skoulidis and Heymach, 2019). LncRNAs play a critical role in maintaining genomic instability (Nair et al., 2020; Jianfeng et al., 2021). Increasing evidence has revealed the prognostic significance of GIRlncRNAs for cancers (Fang et al., 2021; Liang et al., 2021; Maimaiti et al., 2021; Yan et al., 2021). Since NSCLC possesses a poor survival prognosis due to limited diagnosis and treatment (Zappa and Mousa, 2016; Li C. et al., 2019), we developed a prognostic GIRlncSig to support the clinical stratification and treatment decision for NSCLC patients.

In this study, we screened out 1446 GIRlncRNAs for NSCLC by a somatic mutation burden hypothesis-derived computational frame. Functional analysis revealed that these lncRNAs were mainly enriched in nucleoside or ribonucleoside metabolism, cell-cycle checkpoint, nuclear membrane enveloping, and tumorigenesis, which are involved in maintaining genomic instability (Sieber et al., 2003; Deng, 2006; Aird and Zhang, 2015; Lim et al., 2016). TP53 and CSMD3 were the two most frequently mutated genes in NSCLC (Liu et al., 2012), and their mutation status was closely associated with high TMB causing genomic instability and poor clinical prognosis (Zhang et al., 2017; Bernard et al., 2020; Lu et al., 2021; Wen et al., 2021). We further conducted hierarchical clustering analysis and differential analysis of mutation counts and found that GIRlncRNA-clustering GU-like patients were burdened with a higher TMB than GS-like patients. The mutation frequencies of TP53 and CSMD3 genes in GS-like subtype patients were expectedly higher than those in GU-like subtype patients. The survival of GS-like subtype patients was significantly better than that of GU-like subtype patients. Furthermore, we constructed a GIRlncSig encompassing 19 lncRNAs with robust performances, which could predict prognosis independently of other clinicopathological variables and data sets. Among the 19 GIRlncRNA signature, 14 lncRNAs (AC023824.3, AC013287.1, AP000829.1, LINC01611, AC097451.1, AC025419.1, AC079949.2, LINC01600, AC004862.1, AC021594.1, MYRF-AS1, LINC02434, LINC02412, and LINC00337) were risk factors for prognosis, while the other five lncRNAs (LINC01067, AC012645.1, AL512604.3, AC008278.2, and AC089998.1) were protective factors for survival of NSCLC patients. To the best of our knowledge, most lncRNAs we identified here are novel GIRlncRNAs for NSCLC, while some GIRlncRNAs were already reported in lung adenocarcinoma (Geng et al., 2021; Peng et al., 2021; Wu G. et al., 2021). Notably, lncRNA AC023824, AC025419.1, AC079949.2, LINC02412, and LINC00337 were verified as risk factors associated with the OS of LUAD patients (Li R. et al., 2019; Song et al., 2020; Shao et al., 2021; Wu G. et al., 2021; Wu Y. et al., 2021). LINC01600 and LINC02434 were reported as predictors for the prognosis of PCa and HNSCC patients, respectively (Xu et al., 2020; Jiang et al., 2021). Importantly, we performed ROC and calibration analyses to evaluate the GIRlncSig-based risk score and found that it possessed an intact performance with good agreement between the actual survival and predicted survival in 10 years. In contrast, Wang and Geng’s prognostic GIRlncSig only displayed a decreased value of AUCs in 3 years. Therefore, our novel GIRlncSig could provide robust clues for clinical diagnosis and stratification of NSCLC.

Mutations in driver genes are crucial to promoting tumorigenesis and development. NSCLC with positive driver genes possesses high mortality and metastasis risk (Wu Y. et al., 2021; Yuan et al., 2022). We found here that nine driver genes (SOX2, FGFR2, NFE2L2, PTPRD, PTPRT, EGFR, NRG1, MB21D2, and CSMD3) were upregulated. Notably, the expression levels of CSMD3, NFE2L2, and MB21D2 were substantially higher in GU-like samples than those in GS-like samples. NFE2L2 mutation, a major molecular driver of clinical radio resistance (Binkley et al., 2020), was more frequently found in advanced patients to cause a worse prognosis than in patients carrying the wild-type genotype (Sasaki et al., 2010). MB21D2, a key enzyme involved in the cGAS/STING signaling pathway, is also frequently mutated in NSCLC and HNSCC to promote tumor progression (Campbell et al., 2016; Gracilla et al., 2020). The high risk lncRNAs AP000829.1 and LINC01600 from GIRlncSig were positively correlated with MB21D2 expression, which was remarkably upregulated in the high-risk group. Another high-risk lncRNA, AP000829.1, was negatively correlated with NKX2-1. In fact, upregulated AP000829.1 was always accompanied by downregulated NKX2-1 in the high-risk group. NKX2-1 may control lung cancer progression through the induction of DUSP6, an ERK phosphatase, to decrease ERK activity (Ingram et al., 2022). The protective lncRNA, AC008278.2, was positively correlated with the driver gene PTPRT, and this pair was downregulated in the high-risk group. Because PTPRT is an endogenous inhibitor of STAT3 (Sen et al., 2020), loss-of-function mutations in PTPRT resulted in STAT3 hyperactivation to promote the malignancy of NSCLC (Wang et al., 2021). Moreover, the functional genes markedly enriched in the high-risk group mainly involve mismatch repair, base excision repair, nucleotide excision repair, non-homologous end joining, and homologous recombination. These pathways were evidenced to be strongly associated with genomic stability (Majidinia and Yousefi, 2017). Together, these findings suggested that the lncRNAs of our GIRlncSig are remarkably modulated by the NSCLC driver genes, and that their functions are highly consistent with important biological behaviors.

The contextures of the tumor microenvironment, like infiltrated immune cells, stromal cells, cancer stem cells, and TMB, critically determine the progression of cancer (Whiteside, 2008). Hence, pursuing them can help to predict clinical outcomes, guide early diagnosis and improve the therapeutic response (Binnewies et al., 2018). We found here that the novel GIRlncSig could reflect the characteristics of the tumor microenvironment, and GIRlncSig-predicted high-risk patients exhibited features of malignancy with high levels of TMB and mRNAsi, as well as immune, stromal, and ESTIMATE scores. These results accord with the fact that genome instability contributes to neoplasia and metastasis (Bakhoum et al., 2018; Nguyen et al., 2022). Moreover, we found that NSCLC patients from high-risk groups were more sensitive to CTLA4 inhibitors than those from the low-risk group. Collectively, our GIRlncSig may guide the diagnosis and improve the clinical outcome of NSCLC by selecting a subgroup of patients that are more sensitive to this type of immunotherapy.

Two limitations are associated with this study. First, as appropriate GEO datasets were not found, some potential lncRNAs may have been excluded in our GIRlncSig. Hence, more external dataset validation is needed in future studies. Second, the biological functions of twelve newly identified GIRlncRNAs (AC013287.1, AP000829.1, LINC01611, AC097451.1, AC004862.1, AC021594.1, MYRF–AS1, LINC01067, AC012645.1, AL512604.3, AC008278.2, and AC089998.1) are not known yet. Future investigations should elucidate their functions, both *in vitro* and *in vivo*.

## Conclusion

In summary, we constructed a GIRlncSig consisting of 19 GIRlncRNAs. This signature could predict the clinical outcome of NSCLC patients independently of other variables. Moreover, our GIRlncSig could dissect the contextures of the tumor microenvironment and driver genes to guide the diagnosis for improved stratification and individualized treatment of NSCLC patients.

## Data Availability

The datasets presented in this study can be found in online repositories. The names of the repository/repositories and accession number(s) can be found in the article/Supplementary Material.
