# Supplementary material for: Novel GIRlncRNA Signature for Predicting the Clinical Outcome and Therapeutic Response in NSCLC
Source: Front Pharmacol. 2022 Aug 3;13:937531. doi: 10.3389/fphar.2022.937531 (PMC9382191; doi:10.3389/fphar.2022.937531)
Supplement: Supplementary file 5 [file Image9.pdf]

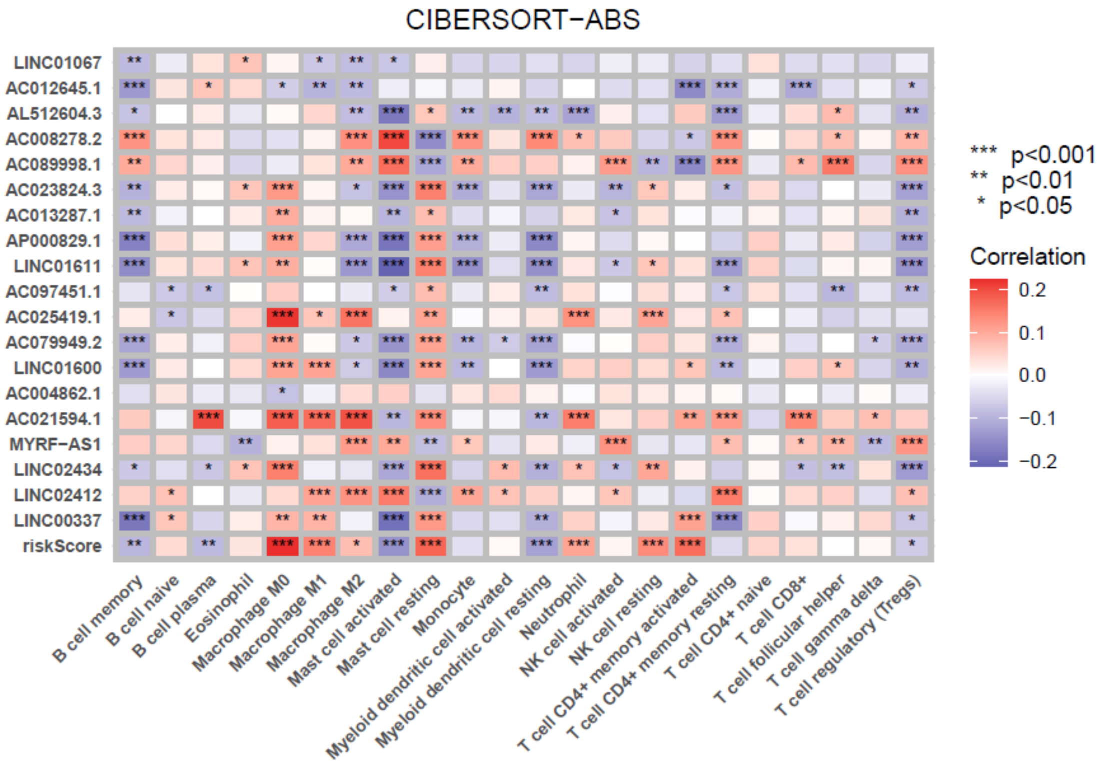


**FIGURE S9** GIRlncRNAs-associated NSCLC-infiltrating immune cells analyzed by the CIBERSORT-ABS package.
