# Supplementary figures and images for: Novel GIRlncRNA Signature for Predicting the Clinical Outcome and Therapeutic Response in NSCLC
Source: Front Pharmacol. 2022 Aug 3;13:937531. doi: 10.3389/fphar.2022.937531 (PMC9382191; doi:10.3389/fphar.2022.937531)

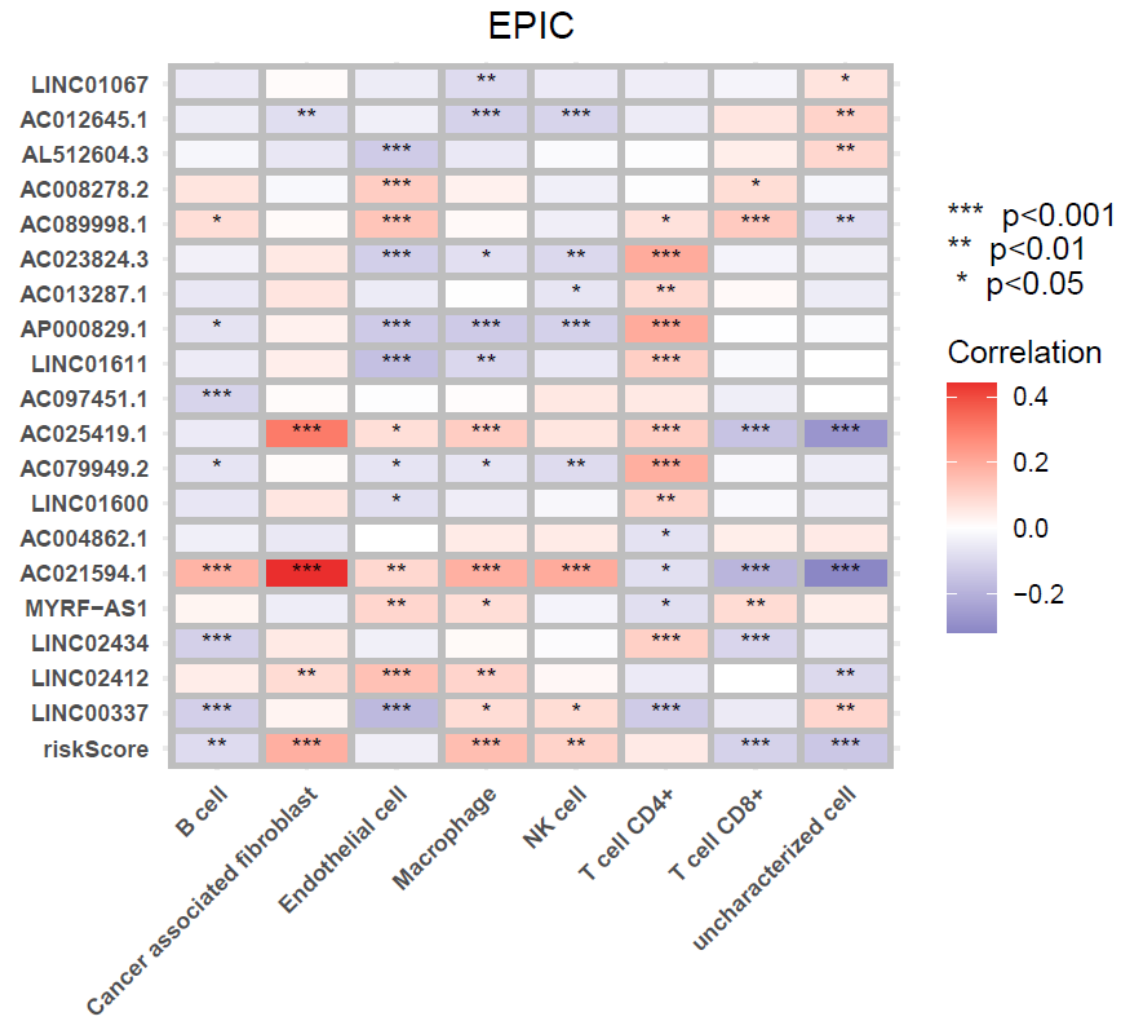

**FIGURE S8** GIRlncRNAs-associated NSCLC-infiltrating immune cells analyzed by the EPIC package.

Supplement: Supplementary file 9 [file Image8.pdf]

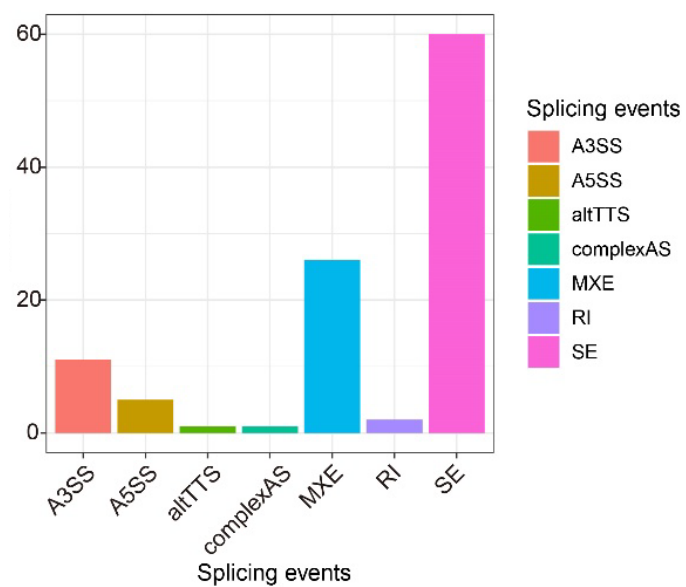

**FIGURE S3** GIRlncRNAs-associated AS events and their statistical analysis.

Supplement: Supplementary file 13 [file Image3.pdf]

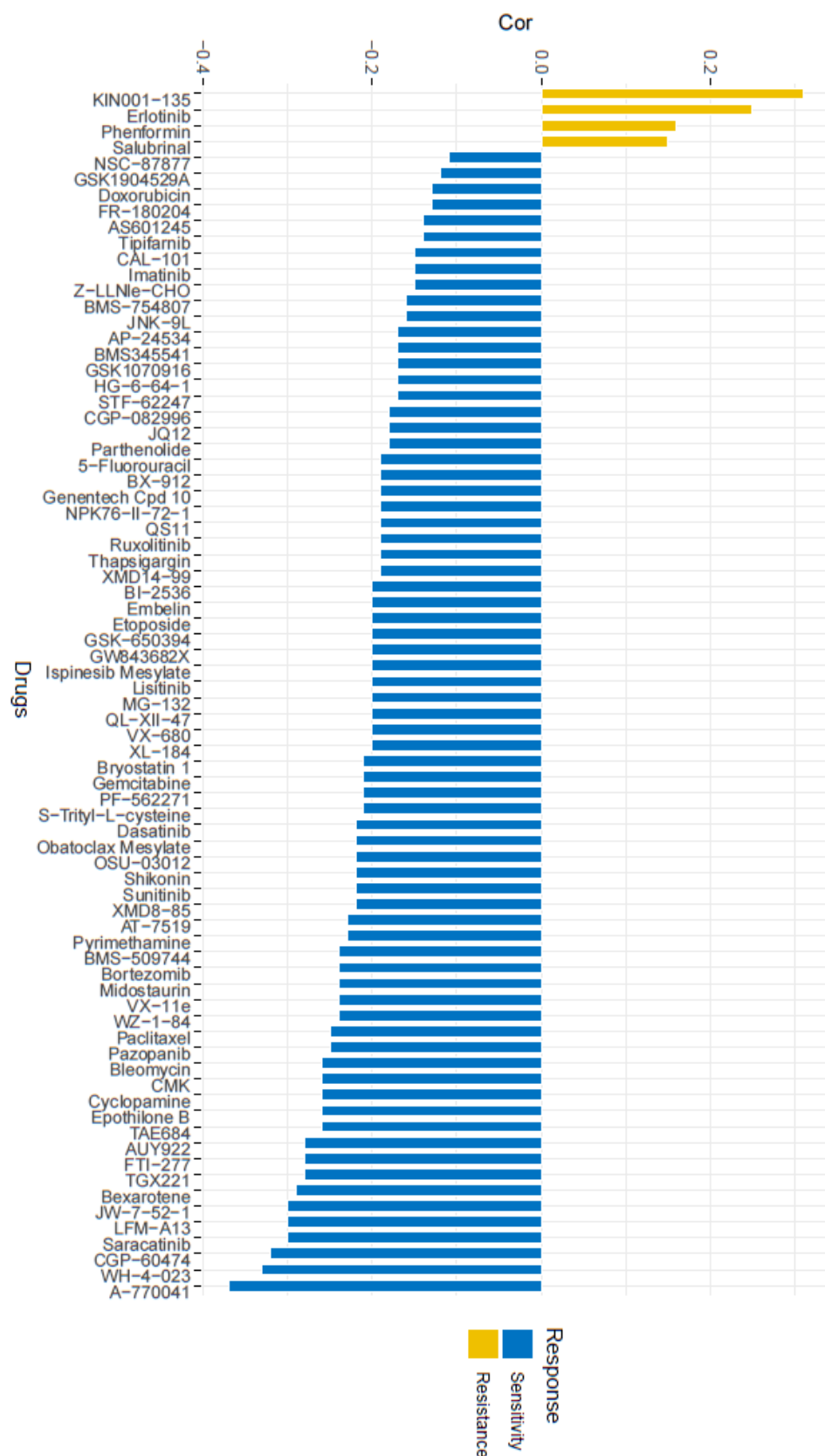

**FIGURE S11** Correlation analysis of GIRlncRNA Signature expression with drug resistance.

Supplement: Supplementary file 17 [file Image11.pdf]

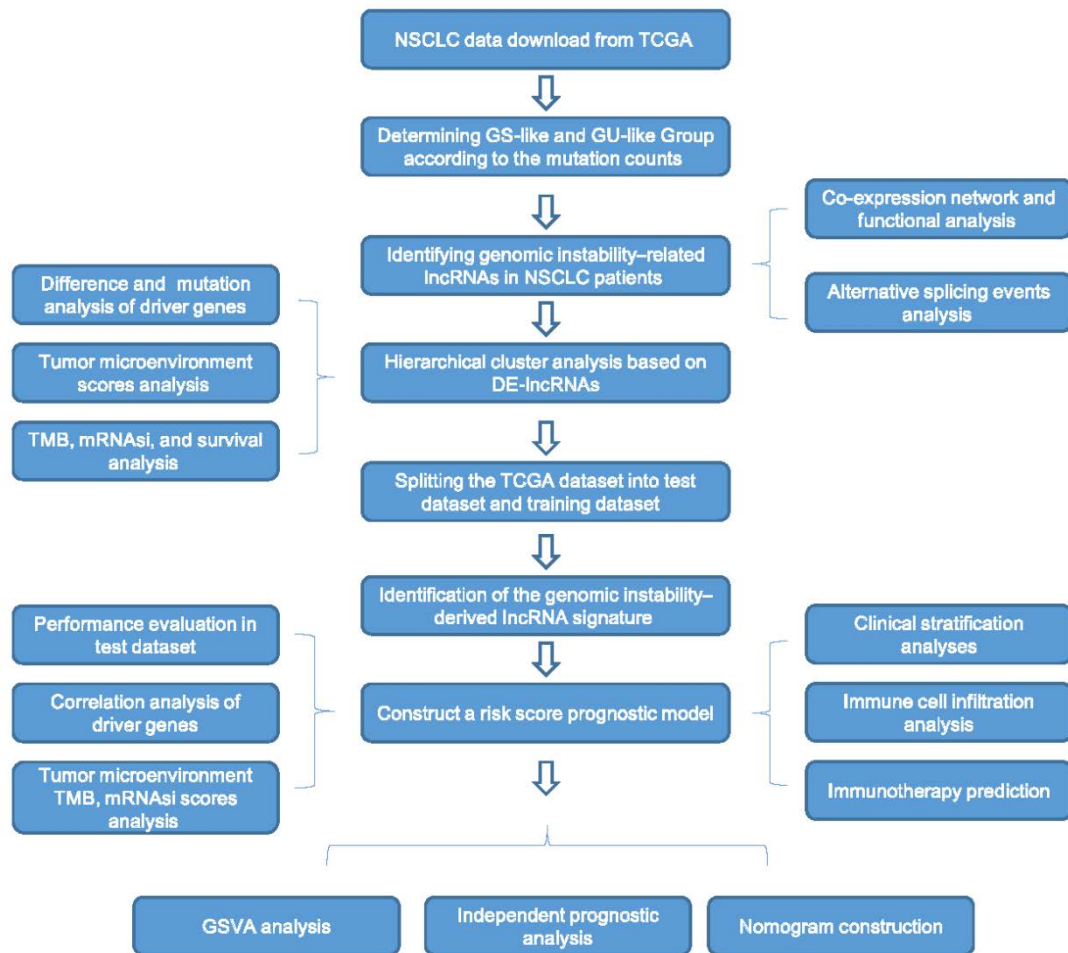

**FIGURE S1** Research roadmap of this study.

Supplement: Supplementary file 25 [file Image1.pdf]
