# Supplementary material for: Novel GIRlncRNA Signature for Predicting the Clinical Outcome and Therapeutic Response in NSCLC
Source: Front Pharmacol. 2022 Aug 3;13:937531. doi: 10.3389/fphar.2022.937531 (PMC9382191; doi:10.3389/fphar.2022.937531)
Supplement: Supplementary file 11 [file Image2.pdf]

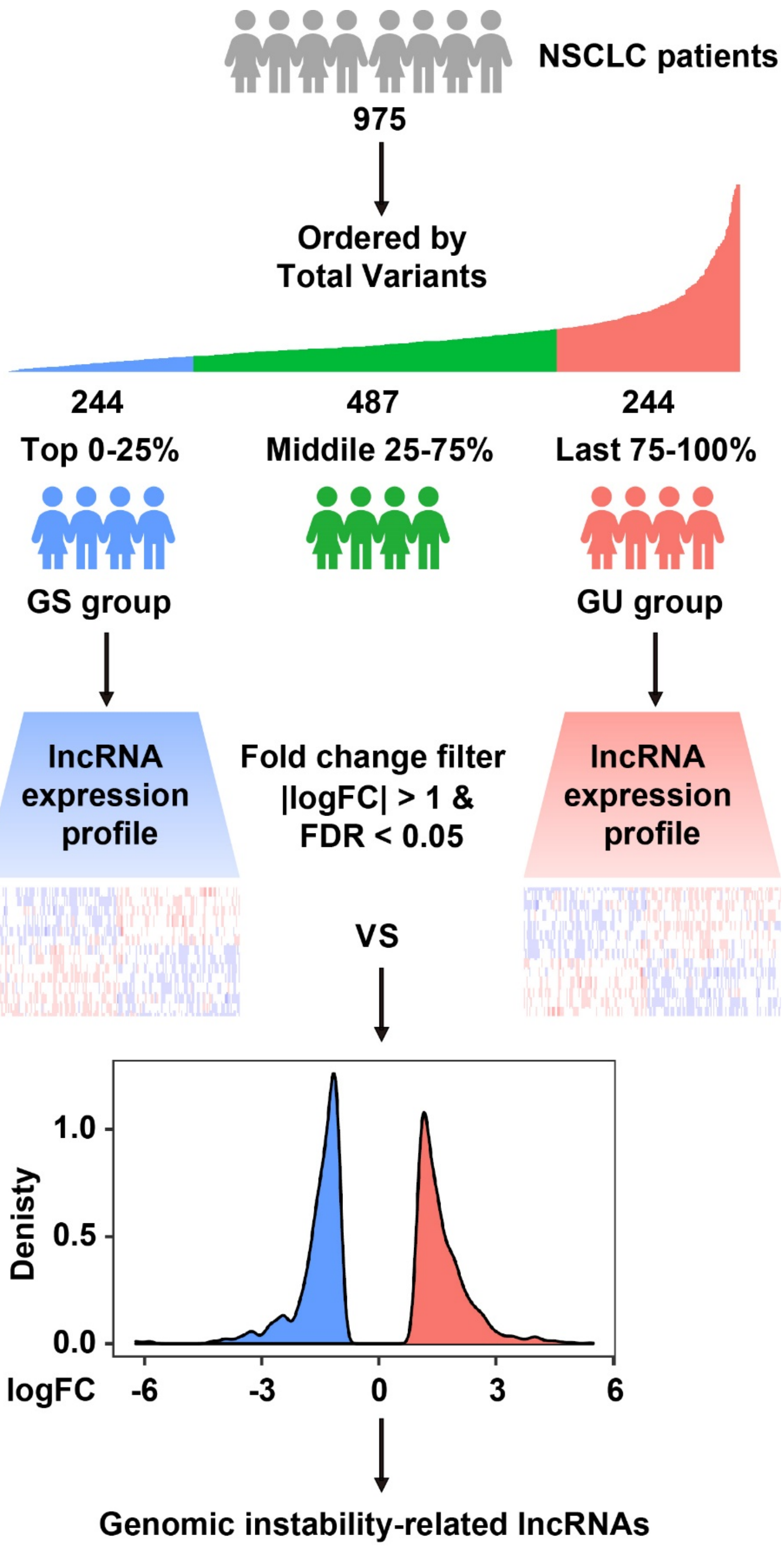

FIGURE S2 Schematic diagram of extracting GIRlncRNAs. 975 NSCLC patients were ordered by the total variants of somatic mutations. The mutator hypothesis-derived computational frame was used to divide the samples into two groups: GU group (the top 25%) and GS group (the last 25%). GIRlncRNAs were extracted by identifying the differential expression lncRNAs between GU and GS groups.
