# Supplementary material for: Novel GIRlncRNA Signature for Predicting the Clinical Outcome and Therapeutic Response in NSCLC
Source: Front Pharmacol. 2022 Aug 3;13:937531. doi: 10.3389/fphar.2022.937531 (PMC9382191; doi:10.3389/fphar.2022.937531)
Supplement: Supplementary file 18 [file Image7.pdf]

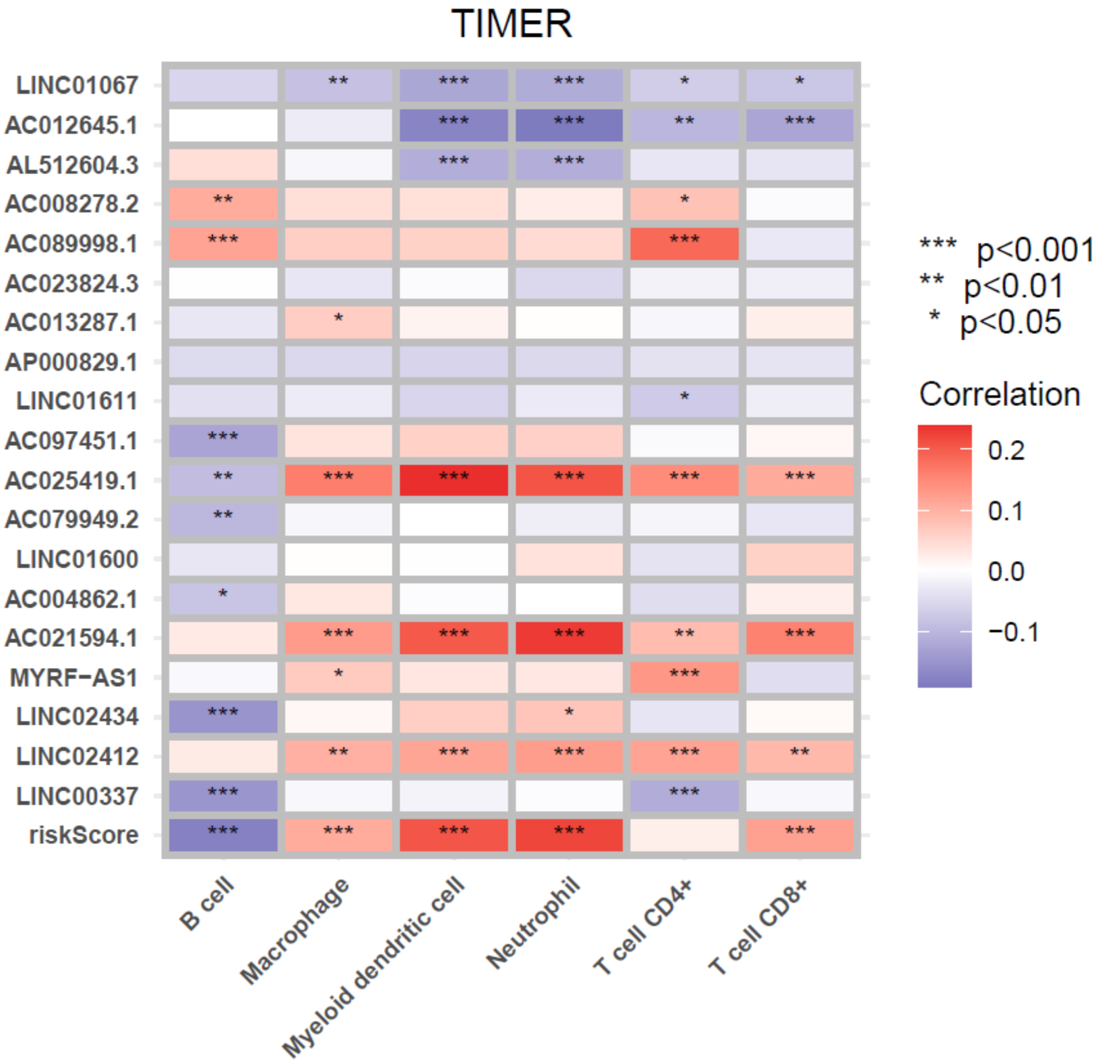

FIGURE S7 GIRlncRNAs-associated NSCLC-infiltrating immune cells analyzed by the TIMER package.
